# On methods for determining solvent accessible surface area for proteins in their unfolded state

**DOI:** 10.1186/1756-0500-7-602

**Published:** 2014-09-03

**Authors:** Shennon Lu, Amy S Wagaman

**Affiliations:** 12010 Blackberry Terrace, 2878 Gaithersburg, MD USA; Department of Mathematics and Statistics, Amherst College, P.O. Box 5000, 01002 Amherst, MA USA

**Keywords:** Proteins, Accessible surface area, Protein folding, Unfolded state, Thermodynamic relationships

## Abstract

**Background:**

There are many different methods for estimating solvent accessible surface area for proteins in their unfolded states. In this article, we compare eight methods, assessing whether or not they lead to different estimates of total accessible surface area as well as their impact on relationships with thermodynamic variables.

**Findings:**

Our results demonstrate that most pairs of compared methods do result in different unfolded estimates of accessible surface area (only four pairs of methods do not yield significantly different estimates). However, we do not see a significant impact on the relationship between accessible surface area and thermodynamic parameters across the different methods.

**Conclusions:**

We advocate the use of the Gong and Rose transition midpoint method for computing solvent accessible surface area due to its computational ease, physical basis, and performance in terms of relationships with thermodynamic parameters.

## Findings

### Background

Protein folding is a process by which a polypeptide transitions from an unfolded state to a native state. While native states are well studied, unfolded states are more difficult to characterize. The hydrophobic effect is the driving force in protein folding wherein hydrophobic groups move away from water into a solvent-shielded hydrophobic core. When folded, solvent accessible surface area (ASA) is lost between the native (folded) and unfolded state. While we can readily compute the ASA for the native state (for example using the algorithm of Lee and Richards [1] or equivalent ones in programs like Chimera [2]), the calculation for ASA for the unfolded protein is more difficult. Several papers have been published claiming to have the best models for calculating the ASA of the unfolded protein or have compared such models or adapted proposed models [3–10]. We want to consider these methods and determine one that is most appropriate for use in a new database of proteins (ACPro, the Amherst College Protein Folding Kinetics Database, available at: https://www.ats.amherst.edu/protein/) organized based on those with folding and unfolding information. Briefly, we explore the literature in which these ASA calculation methods for unfolded states are proposed.

Robertson and Murphy published a review that focused on the relationship between protein stability and structure that was established with the thermodynamic parameters derived from calorimetric and spectroscopic studies and the structural models derived from X-ray crystallography and NMR spectroscopy [11]. As part of their analysis, accessible surface area changes between native and unfolded state for a set of proteins are examined, where the unfolded ASA is based on an Ala-Xaa-Ala extended tripeptide for each amino acid type, where Xaa is a placeholder for that amino acid. Corrections are made for termini effects. The Ala-Xaa-Ala tripeptide method is one of the simplest methods of determining an estimate for unfolded ASA, and was originally proposed by Zielenkiewisz and Saenger [3].

There are many more methods for determining unfolded estimates of ASA. We examine a few methods that are computationally fast and easy to understand because we want to select one for use in a database that will be available to the public. Creamer, Srinivasan, and Rose propose alternatives to the tripeptide model [5, 6]. As alternatives, they propose two models that bracket the expected behavior of an unfolded protein. The first model provides upper bound values on the unfolded ASA. These upper bound values are based on simulated flexible peptides modeled from hard-sphere approximation for ASA and chain dimensions. The use of the hard-sphere approximation results in expanded peptides that explore available conformational space freely, as compared to actual peptides, which experience intramolecular attractive forces, leading to further chain collapse. Consequently, these simulated peptides exclude volume effects and are more expanded than actual unfolded peptides. The second model is for lower bound values of ASA. The lower bound values are modeled from protein fragments excised from fully folded structures. Due to being determined from fragments excised from folded proteins, ASA values in this model will provide a lower bound for unfolded ASA. The conformational behavior of unfolded peptides, thus, lies between the two limits. In their analysis, by comparing the upper and lower bounds to tripeptide models, Creamer et al. argue that the tripeptide models overestimate the area loss [5]. For example, they show that the alanine side chain in the center of an 11-residue, unfolded polyalanyl peptide loses little to no area upon helix formation and a valine side chain gains area in the helix, on average. A tripeptide model would conclude that both alanine and valine side chains lose surface area with helix formation. In 1997, Creamer et al. adjusted the upper bound model when extending it to the backbone case, using the approach from Spolar et al. [4], and stating that this yielded similar values to their previous approach and is less computationally intensive [6]. To compromise between the upper and lower bound models proposed by Creamer et al., other researchers used the average of the two bounds, effectively providing a third model for unfolded ASA [7, 8].

More recently, Gong and Rose proposed a new method to calculate solvent-dependent ASAs of amino acid residues in unfolded proteins [10], which they contrast primarily with that of Creamer et al. [6]. They argue that the method of averaging the ASA residues of the unfolded states between the upper limit and lower limit is unsatisfying because it lacks a rigorous physical basis. Gong and Rose’s own method, on the other hand, is physically based to calculate backbone and side-chain residue surface areas by using data from peptides generated by varying the possible dihedral angles to coincide with allowed regions of conformational space. They use intramolecular hydrogen bond strengths to model solvent-dependent effects by a Boltzmann-weighted distribution of solvent quality through a “hydrogen-bond dial”. When plotted as a function of hydrogen bond strength, the Boltzmann-weighed distribution of conformers describes a sigmoidal curve, with a transition midpoint near -1.5 kcal/mol per hydrogen bond. For the backbone, these midpoint ASA values are similar to Creamer’s upper bounds and in some cases, even exceed the upper bounds set in [6]. The authors argue that this is due to increased flexibility in this new model. Gong and Rose do admit that their model is imperfect because the hydrogen bond dial does not use all possible energetic terms [10]. Due to the “dial”, Gong and Rose provide ASA values when the “dial” is “off” and at the transition midpoint. For terminology, we call the “off” values the upper bounds and we call the transition midpoint values the lower bounds for this method, to be analogous to [6]. Averaging the values at the two bounds yields an average value for Gong and Rose’s proposal.

Finally we examine a more computationally intensive method, ProtSA, proposed by Bernado et al. [9]. The method is made available by a web application [12] (available at: http://webapps.bifi.es/protsa/#Xbernado:2006) and calculates sequence specific protein solvent accessibilities in the unfolded ensemble by simulating the unfolded protein many times and combining the results. In the simulations, the structural model to describe the unfolded conformations representative of the unfolded protein is generated by the Flexible-Meccano algorithm. The analytical software ALPHASURF is applied to calculate atom solvent accessibilities. The researchers report the average ASA for each amino acid over many examples (and simulations) in [9], but the web application allows for non-static values to be generated as well [12].

While this list of methods is not complete (the reader is directed to [9] for a more complete review), we believe it is a representative sample of methods to compare. In this note, we use statistical analysis to compare the ASA values generated by these methods to find significant differences between the methods, if present. We compare the tripeptide method (Ala-Xaa-Ala), Creamer et al. upper bound, lower bound, and average methods, Gong and Rose average and lower bound (transition midpoint) methods, ProtSA static (based on average values) and web server values. For details on computations, please see the Methods section. We also compare the resulting changes in solvent accessible surface area and their relationships with established variables in the literature from [11].

### Unfolded ASA results

To demonstrate the differences between the seven unfolded ASA methods (not including the tripeptide model), we examine the values they assign to individual amino acids in Table 1. It is fairly evident that the individual amino acid values vary a great deal between methods, but we do not know if that variety results in significantly different total unfolded ASA values for proteins. To attain a total unfolded ASA value for each protein, as described in detail in Methods, we assign the values from Table 1 to the corresponding amino acids in each protein or we attain the values from the ProtSA web server, depending on the method, and sum them (after accounting for termini effects).

**Table 1:** **Unfolded surface area coefficients by amino acid for static methods**

Amino acid	Lower creamer	Average creamer	Upper creamer	PROT SA static	Lower gong/rose	Average gong/rose
ALA	66.4	82.95	99.5	73.2	93.8	97.85
ARG	174	196.15	218.3	178.9	209.9	220.1
ASN	102.1	115.2	128.3	109.2	113.1	118.85
ASP	97.3	113	128.7	102.2	126.5	263.6
CYS	81.1	99.3	117.5	88.7	122	126.5
GLN	122.2	142.15	162.1	126	138.7	145.35
GLU	120.7	139.05	157.4	125.9	156.8	161.55
GLY	54.6	65.15	75.7	54.3	67.9	71.4
HIS	118.8	135.65	152.5	129.5	167	171.4
ILE	115.3	137.05	158.8	122.5	158.1	162.6
LEU	116.1	132.25	148.4	131.9	164.1	168.85
LYS	160.8	176.7	192.6	149.9	187	194.8
MET	122	147.65	173.3	134.3	173.8	178.4
PHE	134	153.55	173.1	146.1	188.6	193.35
PRO	102.4	109.5	116.6	100.3	125.8	128.4
SER	83.5	95.9	108.3	76	101.4	106.5
THR	95.9	108.3	120.7	93.3	121.8	127.15
TRP	169.8	180.1	190.4	173.2	226.1	232.65
TYR	148.7	167.25	185.8	156.9	205.7	209.75
VAL	97.7	116.75	135.8	102.2	134.7	139.1

All coefficients are provided. Backbone and sidechain values have been summed to attain one value for each amino acid.

**Figure 1:**
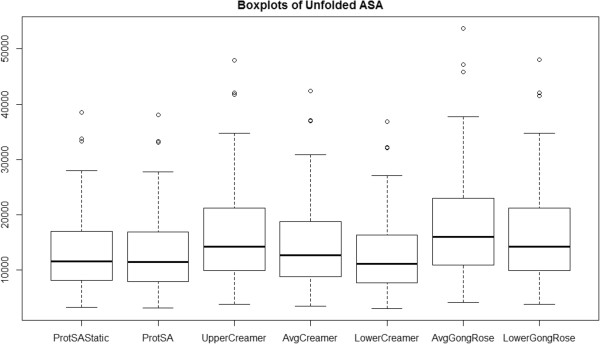
**Boxplots of unfolded solvent accessible surface area.** Unfolded ASA values are provided for comparison across methods for the set of 51 proteins.

Next we examine a series of boxplots showing the total unfolded ASA values across a set of 51 proteins (Figure 1) chosen to align with the data set of [11]. This data set is moderate in size, and naturally, we would like as much data as possible to aid in our selection of an unfolded ASA calculation method. While the data set size may impose some limitations on conclusions, we have not been able to find a larger set with the necessary information in order to expand our analysis. Our analysis is still able to demonstrate method differences and assist us in a decision about method selection for our database.

We note several key characteristics in Figure 1. First, there are a few outliers which are the same proteins under each method (protein databank files (PDBs): 1ABE, 2CAB, 5PEP, 3PSG, 3SIC, and 2ST1). Next, the ProtSA static and ProtSA (web server) distributions look to be very similar, but the ProtSA static values are shifted up a bit relative to the web server values. We do see evidence to confirm what was stated in [10], that the lower bound (transition midpoint) method of [10] results in similar values to those obtained from the Creamer et al. upper bound method from [6]. The lower bound Creamer et al. method seems to give values most similar to those from ProtSA (static and web server).

**Figure 2:**
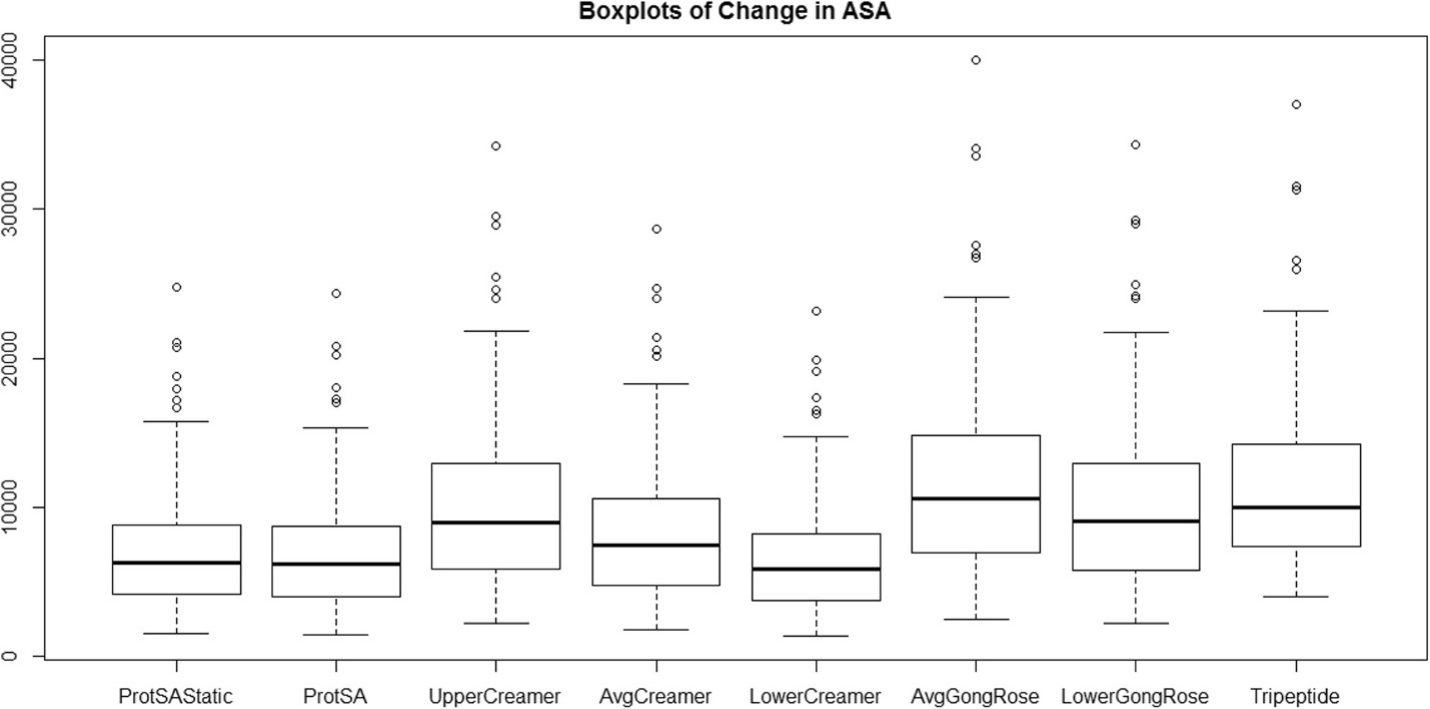
**Boxplots of change in solvent accessible surface area.** Changes in ASA values are provided for comparison across methods including tripeptide results for the subset of 44 proteins.

Next, we examine boxplots of change in ASA across the eight methods (includes previous seven methods and tripeptide values from [11]) as shown in Figure 2. Please see Methods for computational details. The tripeptide (Ala-Xaa-Ala) values do appear to be a little higher than those of upper limit Creamer et al. method (as proposed in [5]), but not by much. This leads to a natural question. Are the differences observed in the boxplots significant? Hence we turn to our statistical analysis.

### Change in ASA results

We computed change in ASA values from the unfolded to folded state after acquiring folded ASA estimates using Chimera [2]. For details on computations, please see Methods. Paired t-tests to look for differences in mean change in ASA values were performed to address whether or not the differences observed in the boxplots are significant (similar results are obtained if such an analysis is performed on just the mean unfolded ASA values due to the only difference in the values being a distinct constant shift for each protein) with adjustments on determining significance due to multiple testing. This analysis and all subsequent analyses are performed on the subset of 44 proteins where the protein size matched the number of residues value from the review work of [11] so that comparable values were being compared. The paired t-tests indicated that only four contrasts (pairs of methods) resulted in an insignificant result. The insignificant contrasts were between the tripeptide and average Gong/Rose methods, the tripeptide and lower Gong/Rose methods, the tripeptide and upper bound Creamer et al. methods, and the lower Gong/Rose and upper bound Creamer et al. methods. All other pairs of methods resulted in statistically significantly different mean change in ASA values for the proteins examined. The ProtSA static change in ASA values were about 225 units above the ProtSA web server change in ASA values per protein, on average, so this was a significant difference despite their similarity in the boxplots. As many of the methods result in significantly different changes in ASA per protein, we need to determine what method we want to use in the database, ACPro. Next we consider which method (if any) is “best” relative to the performance of the tripeptide method in the relationships with changes in ASA examined in [11], as the newer methods have a stronger physical basis.

To set a baseline threshold of performance, we compute the R-squared value (from a simple linear regression) between the tripeptide change in ASA value on the 44 protein subset and each variable examined in a relationship with change in ASA in [11]: number of residues (Nres), heat capacity change upon unfolding (ΔC_p_), enthalpy of unfolding at 60 degrees C (ΔH(60)) and at 100 degrees C (ΔH*), and entropy of unfolding at 60 degrees C (ΔS(60)) and at 112 degrees C (ΔS*). Then, we compute R-squared values from regressions using the other method’s change in ASA values and the same variables. The resulting R-squared values are provided in Table 2.

**Table 2:** **R-squared values using change in ASA values**

Response variable	Tripeptide R^2	ProtSA static	ProtSA	Upper creamer	Avg creamer	Lower creamer	Avg gong/rose	Lower gong/rose
Nres	*.9945*	.9936	.9932	**.9958**	**.9945**	.9921	.9934	**.9954**
∆C_p_	*.7857*	**.7801**	.7797	**.7815**	.78	.7772	.7777	**.7838**
∆H(60)	*.8034*	.7909	**.7951**	.7944	.7943	.7937	**.8087**	**.7982**
∆S(60)	*.7707*	.7665	.7663	.7655	.7661	**.7666**	**.7776**	**.7685**
∆H*	*.937*	.9263	.9296	**.93**	.9292	.9275	**.9365**	**.9331**
∆S*	*.9344*	.928	.929	**.9295**	.929	.9278	**.9343**	**.9321**

R-squared values are from regressions using each unfolded surface area method to predict the six different response variables from Robertson and Murphy [11]: number of residues (Nres), heat capacity change upon unfolding (∆C_p_), enthalpy of unfolding at 60 degrees C (∆H(60)) and at 100 degrees C (∆H*), and entropy of unfolding at 60 degrees C (ΔS(60)) and at 112 degrees C (∆S*). Values in bold are either improvements, ties, or in the closest three to the performance of the tripeptide method in terms of R-squared values. Tripeptide reference values are in italics.

Based on the results in Table 2, we note that between methods where the change in ASA is identified as being in the best three predictors for each response variable, the differences in R-squared values (which are equivalent to slight differences in correlations), are not large enough to be statistically significant. Indeed, across most of the methods (not even restricting ourselves to the three strongest relationships), this would be the case. The method that most closely matches the performance of the tripeptide method in terms of these relationships is the lower bound (transition midpoint) Gong/Rose method.

Based on our results, the freely available ACPro database containing protein folding kinetics information makes use of the lower bound (transition midpoint) Gong/Rose method for computing unfolded ASA for the proteins reported. The rationale is as follows: the method has a strong physical basis as provided in [10], is not computationally intensive, and termini effects are easily dealt with. While this method yields significantly different estimates of ASA than some of the other methods, it does not suffer in terms of its performance in key relationships with thermodynamic variables previously studied.

## Conclusions

In conclusion, we compare eight different methods of computing change in solvent accessible surface area for proteins, by focusing on different methods of computing the unfolded solvent accessible surface area. We found that while most methods do generate statistically significantly different change in ASA values, there are not significant differences in how well the resulting change in ASA values relate to other thermodynamic parameters in already established relationships. Based on these findings, we chose a method for computing unfolded surface area for use in the ACPro database on protein folding kinetics – the transition midpoint (lower bound) method from [10].

## Methods

### Data and solvent accessible surface area generation

In order to compare methods, a suitable set of proteins was needed. Due to the variety of relationships with change in surface area values studied in [11] we decided to use the same protein set. While the data set is modest in size, it is able to provide a baseline comparison for the other ASA generation methods in the unfolded state, and we can see how well they replicate the results presented in [11]. Of the 53 proteins in [11], we obtained the PDBs for 51 of the proteins from RCSB [13] for which we could compute unfolded surface area values. We left out PDBs 2WRP and 1STF due to data processing issues; we were unable to obtain all necessary values for those two proteins. Then, comparing the protein sizes reported in [11] and the values we obtained, we had a subset of 44 proteins on which the sizes matched. We perform analyses on both the set of 51 proteins and the subset of 44 where size matched. The reason for using the subset is that the review did not report unfolded ASA, instead reporting only change in ASA using the tripeptide (Ala-Xaa-Ala) method for unfolded ASA, so we will examine the impact of the tripeptide method using change in ASA in order to be sure that our data is appropriate for the comparison. For the subset of 44 proteins, we recorded the change in ASA values reported in Table three of [11], as well as a subset of other thermodynamic variables from Table 2 of [11], including number of residues (Nres), heat capacity change upon unfolding (ΔC_p_), enthalpy of unfolding at 60 degrees C (ΔH(60)) and at 100 degrees C (ΔH*), and entropy of unfolding at 60 degrees C (ΔS(60)) and at 112 degrees C (ΔS*) to use in our comparison.

For the larger set of 51 proteins, we obtained unfolded ASA values using the following seven methods: Creamer et al. upper bound, lower bound, and average methods [5, 6], Gong and Rose average and lower bound (transition midpoint) methods [10], ProtSA static (based on average values) [9] and web server values [12]. All methods except the web server for ProtSA give static (constant) values for each amino acid, which are reported in Table 1.

To compute the unfolded surface areas for each of the six static methods, we wrote code in R [14] that took as an input the amino acid sequence in each protein written in standard three letter code, and assigned the corresponding value from Table 1 for each method to each amino acid. We had some minor concerns due to termini effects. Notably, in [6], the first and last three residues of each chain were excluded from the ASAs to avoid these effects. We also excluded the first and last three residues for all peptide chains in all methods to have consistent calculations. Thus, we summed values to attain a total unfolded ASA for each protein, leaving off the first and last three residues in each chain.

For the ProtSA web server values, we submitted jobs to the ProtSA server (http://webapps.bifi.es/protsa/#Xbernado:2006) in batches using the default settings of 1.4 Å for solvent radius and using 2000 unfolded conformations to generate results. We obtained results via email as the server processed them, and then recorded the unfolded ASA values. The ProtSA server reports give a value for each amino acid, which we totalled to obtain the value for each protein, after accounting for termini effects (ignoring first and last three residues) as with the static methods.

To enable a comparison between the tripeptide model, for which we only had change in ASA values, and our other models, we used Chimera [2] to generate folded surface area estimates for each protein in the data set, and then computed changes in surface area from the Chimera estimate to the seven different unfolded estimates we generated via subtraction. We note that the change in ASA values in [11] are stated to have been corrected for termini effects, so we believe this makes the sets of change in ASA values comparable between the seven we generated and the tripeptide values recorded from the review [11].

### Statistical analysis

In order to compare the differences in unfolded ASA and changes in ASA, for the 51 proteins for which we had seven different unfolded surface areas, and the subset of 44 proteins for which we had eight different changes in surface areas, a repeated measures ANOVA would be ideal. However, the sphericity condition failed, so instead we compared all possible pairs of methods with paired t-tests with adjusted p-values due to multiple testing. After comparing the methods in terms of unfolded ASA and change in ASA obtained, we also performed simple linear regression analyses on the subset of 44 proteins for which we had additional data from [11] where our protein size matched what was reported for chain length. Our aim was to find out if any of the methods could obtain stronger relationships with the reported thermodynamic variables than those when the tripeptide (Ala-Xaa-Ala) ASA method was employed.

## Availability and requirements

**Project name:** None

**Project home page:** None

**Operating system(s):** Platform independent

**Programming language:** R

**Other requirements:** None

**License:** None

**Any restrictions to use by non-academics:** None

## Availability of supporting data

Most data used is from [11] with PDB files from RCSB [13] used to calculate unfolded and folded surface area estimates. Unfolded state surface area computations were performed in R [14] based on the protein structures obtained from RCSB or from ProtSA server results. Chimera [2] was used for folded state surface area computations. See Methods for details.

## Abbreviations

ASA: Accessible surface area
PDB: Protein databank file.
